# *MdAIL5* overexpression promotes apple adventitious shoot regeneration by regulating hormone signaling and activating the expression of shoot development-related genes

**DOI:** 10.1093/hr/uhad198

**Published:** 2023-10-10

**Authors:** Kai Liu, An Yang, Jiadi Yan, Zhaolin Liang, Gaopeng Yuan, Peihua Cong, Liyi Zhang, Xiaolei Han, Caixia Zhang

**Affiliations:** Apple Breeding, Chinese Academy of Agricultural Sciences Research Institute of Pomology, Xingcheng 125100, China; Key Laboratory of Horticultural Crops Germplasm Resources Utilization, Ministry of Agriculture and Rural Affairs of the People's Republic of China, Xingcheng 125100, China; Shandong Academy of Grape, Shandong Academy of Agricultural Sciences, Jinan 250100, China; Apple Breeding, Chinese Academy of Agricultural Sciences Research Institute of Pomology, Xingcheng 125100, China; Key Laboratory of Horticultural Crops Germplasm Resources Utilization, Ministry of Agriculture and Rural Affairs of the People's Republic of China, Xingcheng 125100, China; Apple Breeding, Chinese Academy of Agricultural Sciences Research Institute of Pomology, Xingcheng 125100, China; Key Laboratory of Horticultural Crops Germplasm Resources Utilization, Ministry of Agriculture and Rural Affairs of the People's Republic of China, Xingcheng 125100, China; Apple Breeding, Chinese Academy of Agricultural Sciences Research Institute of Pomology, Xingcheng 125100, China; Key Laboratory of Horticultural Crops Germplasm Resources Utilization, Ministry of Agriculture and Rural Affairs of the People's Republic of China, Xingcheng 125100, China; Zhengzhou Fruit Research Institute, Chinese Academy of Agricultural Sciences, Zhengzhou 450009, China; Apple Breeding, Chinese Academy of Agricultural Sciences Research Institute of Pomology, Xingcheng 125100, China; Key Laboratory of Horticultural Crops Germplasm Resources Utilization, Ministry of Agriculture and Rural Affairs of the People's Republic of China, Xingcheng 125100, China; Apple Breeding, Chinese Academy of Agricultural Sciences Research Institute of Pomology, Xingcheng 125100, China; Key Laboratory of Horticultural Crops Germplasm Resources Utilization, Ministry of Agriculture and Rural Affairs of the People's Republic of China, Xingcheng 125100, China; Apple Breeding, Chinese Academy of Agricultural Sciences Research Institute of Pomology, Xingcheng 125100, China; Key Laboratory of Horticultural Crops Germplasm Resources Utilization, Ministry of Agriculture and Rural Affairs of the People's Republic of China, Xingcheng 125100, China; Apple Breeding, Chinese Academy of Agricultural Sciences Research Institute of Pomology, Xingcheng 125100, China; Key Laboratory of Horticultural Crops Germplasm Resources Utilization, Ministry of Agriculture and Rural Affairs of the People's Republic of China, Xingcheng 125100, China

## Abstract

Adventitious shoot (AS) regeneration is a significant factor in the genetic transformation of horticultural plants. It is also a noteworthy approach to their vegetative propagation. AS regeneration remains highly dependent on the genotype or maturity of explants. We here found that the AS regeneration abilities of apple leaves were positively correlated with *MdAIL5* expression. *MdAIL5* overexpression dramatically increased AS regeneration efficiency. Notably, *MdAIL5* overexpression could restore the AS formation ability of explants to a certain extent, which was lost with an increase in maturity. Endogenous hormone detection revealed that *MdAIL5* overexpression changed the contents of auxin, cytokinin (CK), and other hormones in apple leaves. Transcriptome analysis revealed that many genes related to auxin, CK, and brassinolide signaling pathways were significantly and differentially expressed between *MdAIL5*-overexpressing transgenic apple and wild-type apple plants. Yeast one-hybrid assays, the electrophoretic mobility shift assay, and the dual-luciferase reporter assay revealed that MdAIL5 directly binds to *MdARF9* and *MdHB14* promoters and positively affects their expression. We here established a model of MdAIL5 regulating AS formation, which acts as a theoretical basis for facilitating genotype- or explant maturity-independent AS regeneration in the future.

## Introduction

Plants undergo self-repair or their damaged tissues or structures are replaced during regeneration, and this process allows plants to adapt to the environment [1–3]. Plant regeneration is based on totipotency or pluripotency, which reflects the high flexibility or plasticity of plant cells [4–6]. Plant regeneration can be divided into somatic embryogenesis and *de novo* organogenesis [7]. Among *de nov*o organogenesis, adventitious shoots (ASs) play a crucial role in genetic transformation. Therefore, increasing research attention has been directed toward AS regeneration [8–10].

After nearly 2000 years of domestication and cultivation, apple has been cultivated in most countries and regions globally and is popular with consumers [11, 12]. With the release of several high-quality apple genomes, molecular-assisted selection (MAS) technology has been gradually applied to apple breeding to increase its efficiency [11, 13, 14]. Gene function research is the basis of MAS technology, and apple genetic transformation is the main method used in gene function research. The leaf disk method is mainly used to genetically transform apple, and therefore an efficient AS regeneration system dictates the success of this transformation [10, 15]. For many plants, including apple, AS formation efficiency is highly dependent on the genotype or explant maturity [10, 15–19]. For example, ‘Gala’ and ‘Jonagold’, which are widely used in genetic transformation of apple, have relatively high AS regeneration efficiencies [20], whereas ‘Fuji’ has an extremely low AS regeneration efficiency, which makes successful genetic transformation difficult [21]. Mao *et al*. [10] clarified the AS regeneration ability of several common apple rootstocks. They found that the AS regeneration efficiencies of *Malus prunifolia* (MP) and M26 was >4 times of that of T337 under the same culture conditions. Leaf maturity is another decisive factor for AS formation in apple. Generally, the greater the leaf maturity, the poorer the ability of cells to divide, the slower the growth, and the greater the difficulty of AS regeneration [18, 19, 22]. The low transformation efficiency (0–3%) limited the application of transgenic and gene editing technologies in apple.

The interaction between auxin and cytokinin (CK) determines cell fate transitions during plant regeneration [23]. A high CK concentration mediates the loss of root meristem characteristics in the callus, enabling the callus to gain shoot regeneration ability [23, 24]. Consistently, many genes related to CK biosynthesis and signaling pathways are involved in AS regeneration. The receptors of CK signaling, *Arabidopsis cytokinin-receptor histidine kinases* (*AHK*s), regulate AS regeneration by activating *WUS* expression while inhibiting *WOX5* expression [25, 26]. *Type-B ARR* (*B-ARR*) and *Type-A ARR* (*A-ARR*) positively and negatively regulate the CK response, respectively [27]. Correspondingly, inhibiting the expression of *B-ARR*s and overexpressing *A-ARRs* can reduce the AS regeneration efficiency [28]. Cytokinin dehydrogenase/oxidase (CKX) enzymes irreversibly degrade the excess of CK to regulate its level [29]. Accordingly, *CKX1*-overexpressing plants exhibit lower CK content and AS regeneration efficiency [30]. In apple, MdWOX11 can downregulate *MdCKX5* expression to suppress AS formation [10]. Auxin also plays crucial roles in AS regeneration. The main mediators of auxin signaling, such as indole-3-acetic acids (IAAs), small auxin-upregulated RNAs (SAURs), and AUXIN RESPONSE FACTORs (ARFs), are key drivers of AS regeneration. For example, the *IAA14* gain of function mutant is defective in callus formation and AS regeneration [31]. ARF5 can directly bind *SHOOT MERISTEMLESS* (*STM*) and *CYTOKININ RESPONSE FACTOR 2* (*CRF2*) promoters and activate their expression to promote callus formation and AS regeneration [32]. The AS regeneration efficiency of *zmsaur15* (maize *SAUR15* deletion mutant) was five times higher than that of the wild-type (WT) plant [33]. In addition， Brassinolide (BR), ethylene (ETH), and gibberellin (GA) signaling-related genes can also regulate AS formation, probably in connection with auxin and CK signaling [8, 15, 34].

Conventionally, hormone-based *de novo* organogenesis is cumbersome and time-consuming as well as relying heavily on the experience of operators [16]. Many studies have recently unraveled the molecular basis of plant organogenesis and identified several regeneration-promoting key genes, thereby significantly improving the regeneration efficiency of many plant species. For instance, *AtWUS* overexpression greatly improved the AS regeneration efficiency of *Coffea canephora*, *Gossypium hirsutum*, and tobacco [35–39]. The KNOX homeodomain transcription factor STM prevents the differentiation of meristematic cells [40]. The transformation efficiency of maize *STM* homolog *Knotted1* (*ZmKn1*)-overexpressing citrus increased 3–15 times. The transformation efficiency of *ZmKn1*-overexpressing tobacco increased 3 times. BBM, an AP2/ERF family transcription factor, can promote cell proliferation and ectopic embryo formation in plant somatic embryogenesis and organogenesis [41]. Overexpression of native and heterologous *BBM* genes improves the transformation efficiency of various plant species [42–45]. BBM belongs to the AIL family of transcription factors, which plays a critical role in plant regeneration. In a previous study, we analyzed the expression change of AIL family members during AS regeneration in apple, and the results revealed that *AINTEGUMENTA-LIKE 5* (*AIL5*) (MD13G1252700) was most significantly upregulated, exceeding *BBM1* [21]. Phylogenetic analysis of *Arabidopsis* and apple AILs revealed that MdAIL20 (MD13G1252700) was the predicted ortholog of AtAIL5. To facilitate the functional analysis of MdAIL20 (MD13G1252700), we renamed MdAIL20 (MD13G1252700) as MdAIL5 in the current study. In *Arabidopsis*, AIL5 can induce the expression of the characteristic genes *PLT1* and *PLT2* of the root meristem and the characteristic factors *CUP-SHAPED COTYLEDON 1* (*CUC1*) and *CUC2* of AS regeneration, thus endowing the callus with AS regeneration ability [46]. However, the effect of *AIL5* on AS regeneration in apple needs to be studied further.

For the current study, we classified the AS regeneration ability of different apple cultivars and rootstocks. We further demonstrated that *MdAIL5* overexpression promotes AS formation in apple. Changes were observed in the transcription level of *MdAIL5*-overexpressing (*MdAIL5*-OE) apple lines through RNA-seq and in the hormone content of these lines through liquid chromatography–tandem mass spectrometry (LC–MS/MS). Finally, we demonstrated that *MdARF9* and *MdHB14* act downstream of MdAIL5 to promote AS formation. Our results revealed the mechanism through which MdAIL5 promotes AS formation in apple and provide a theoretical basis for the further application of MdAIL5 to help overcome the recalcitrant transformation of apple.

## Results

### Adventitious shoot regeneration ability varies for different apple genotypes

**Figure 1:**
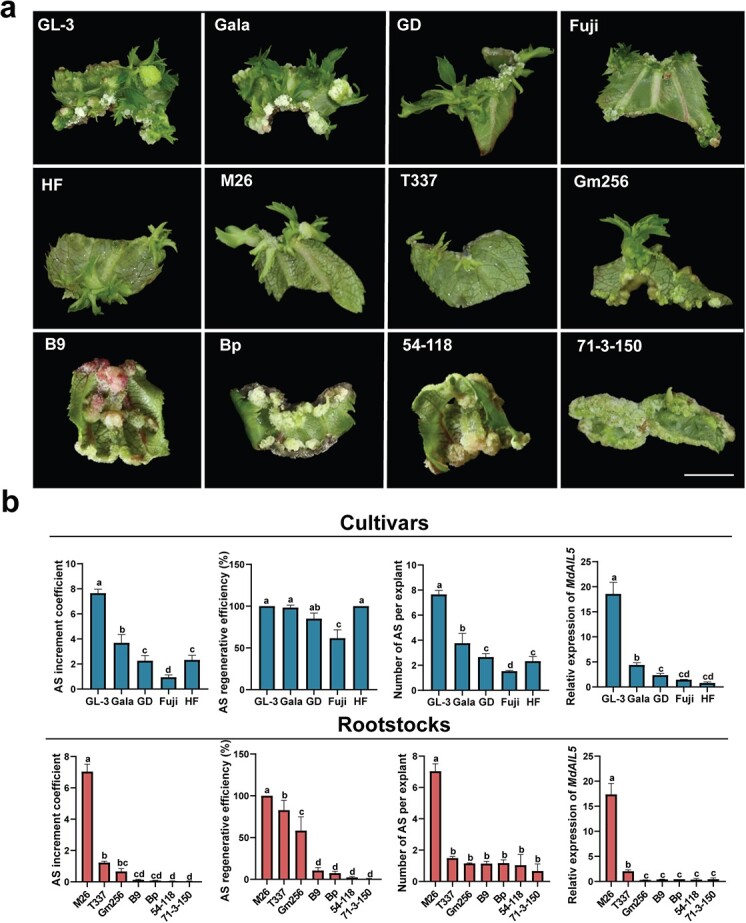
Analysis of AS regeneration from the leaves of different apple genotypes. **a** Morphology of AS regenerated from leaves in ‘GL-3’, ‘Gala’, ‘Golden Delicious’ (GD), ‘Hanfu’ (HF), ‘Fuji’, M16 × M9 (M26), Gm256, M9-T337 (T337), B9, BP-176 (Bp), 54-118, and 71-3-150. Scale bar = 0.5 cm. **b** Quantification parameters of AS regeneration ability and relative expression of *MdAIL5* of four apple cultivars. **c** Quantification parameters of AS regeneration ability and relative expression of *MdAIL5* of seven apple rootstocks. The data were collected from three independent experiments, with each experiment conducted using 60 explants. Error bars = standard deviation. The same lowercase letter indicates no significant difference at *P* < .05, Duncan’s multiple range test.

In the current study, we tested the AS regeneration ability of four major apple cultivars and seven apple rootstocks in the same regenerative medium (2 mg/l TDZ and 0.5 mg/l NAA). The AS increment coefficient, AS regeneration efficiency, and number of ASs per explant for ‘Gl-3’ and M26 were significantly higher than those for other cultivars and rootstocks. The AS regeneration ability of the tested apple cultivars was in the following order (high to low): ‘Gl-3’ > ‘Gala’ > GD > HF > ‘Fuji’ (Fig. 1a and b). The AS regeneration ability of the tested apple rootstocks was in the following order (high to low): M26 > T337 > Gm256 > B9 > Bp > 54-118 > 71-3-150 (Fig. 1a–c). In our previous study, we found that many *MdAIL*s were upregulated during AS regeneration [21]. We here detected the expression of the six most significantly upregulated *MdAIL*s in the leaves of these cultivars and rootstocks after 5 weeks of regenerative culture. The results showed that *MdAIL5* was most significantly upregulated during AS regeneration and its mRNA level and AS regeneration ability were positively correlated (Fig. 1b and c, Supplementary Data Fig. S1). Therefore, MdAIL5 was speculated to play a crucial role in apple AS regeneration.

### *MdAIL5* overexpression enhances adventitious shoot regeneration in apple

**Figure 2:**
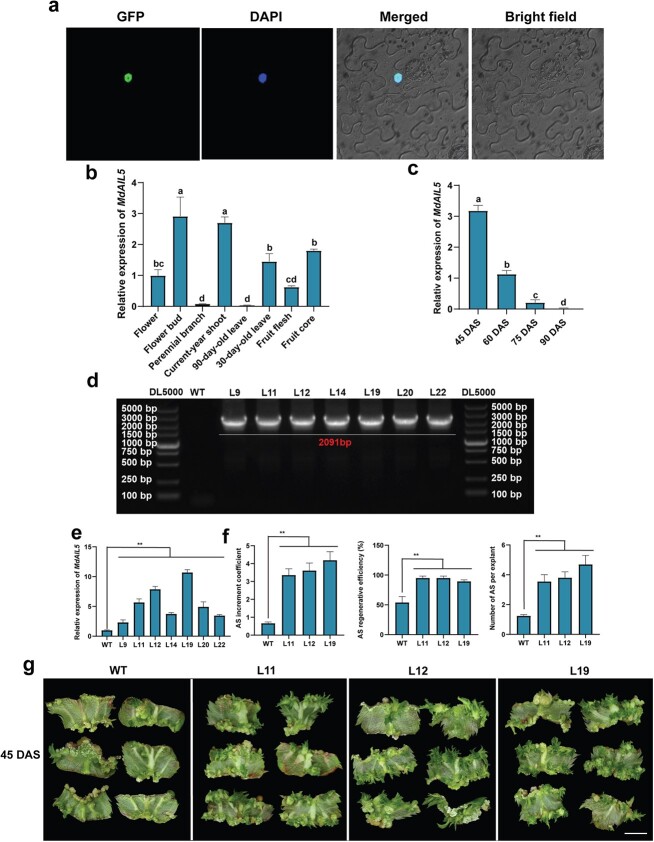
*MdAIL5* overexpression enhanced AS regeneration in apple. **a** Subcellular localization of MdAIL5. Scale bar = 100 μm. **b***MdAIL5* expression levels in different apple tissues. **c***MdAIL5* expression levels of apple tissue culture seedlings on different subculture days. Error bars = standard deviation. The same lowercase letter indicates no significant difference at *P* < .05, Duncan’s multiple range test. **d** DNA detection of *MdAIL5*-OE lines. **e***MdAIL5* expression levels in *MdAIL5*-OE lines and WT. **f** Quantification parameters of AS regeneration ability of *MdAIL5*-OE lines and WT. Error bars = standard deviation. ^**^P < .01 (Student’s *t*-test). **g** Morphology of AS regenerated from leaves of *MdAIL5*-OE lines cultured on MS + 0.1 mg/l TDZ compared with untransformed WT plants. Scale bar = 0.5 cm.

The subcellular localization of MdAIL5 was analyzed through transient expression in tobacco leaf epidermal cells. The fluorescence signal of MdAIL5-GFP was completely fused with that of the DAPI-stained nucleus, indicating that *MdAIL5* was expressed and functional in the nucleus (Fig. 2a). To test the role of *MdAIL5* in AS regeneration, we obtained seven *MdAIL5*-OE lines by using an *Agrobacterium*-mediated method and detected *MdAIL5* mRNA overexpression in the leaves of the transgenic apple lines (Fig. 2d and e).

Lines L11, L12, and L19 with the highest *MdAIL5* expression were selected for subsequent experiments (Fig. 2c). On the commonly used regeneration medium (2 mg/l TDZ and 0.5 mg/l NAA), visual evaluation indicated that the AS regeneration efficiency of the transgenic apple lines was clearly increased. However, because all *MdAIL5*-OE lines and WT plants had regenerated many shoots and these shoots were constantly propagating, conducting quantitative statistics was difficult. To perform quantitative statistics on AS regeneration ability, we further tested *MdAIL5*-OE lines and WT plants on MS medium with a low CK concentration (0.1 mg/L TDZ), and the AS regeneration phenotypes are presented in Fig. 2g. The AS increment coefficient, AS regenerative efficiency, and number of ASs per explant for L11, L12, and L19 were significantly higher than those for ‘GL-3’ apple (Fig. 2f and g).

**Figure 3:**
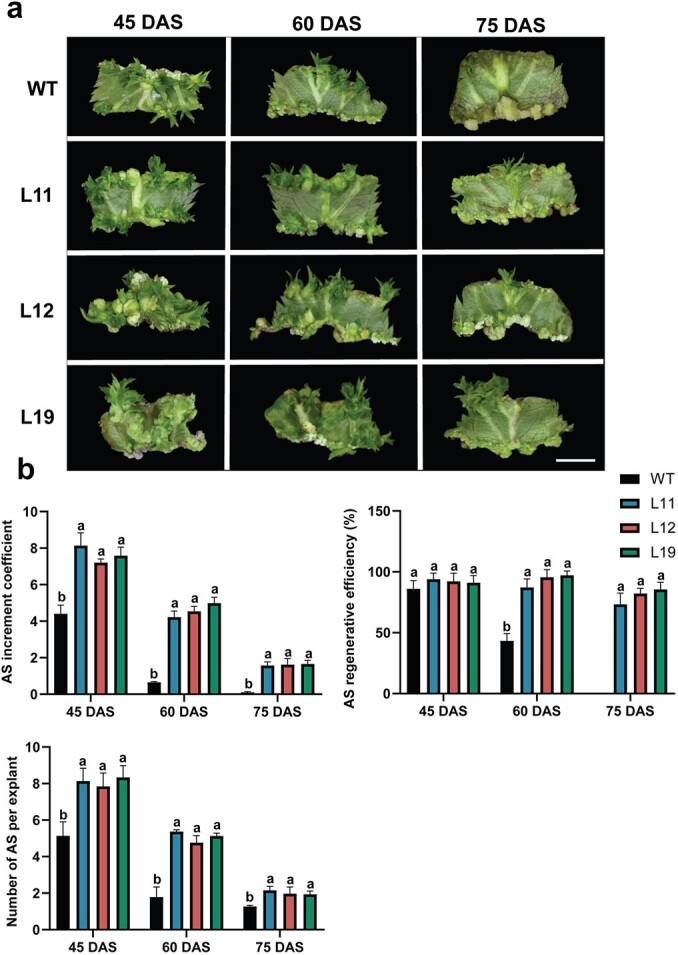
**a** Morphology of ASs regenerated from the leaves of *MdAIL5*-OE lines and WT plants on different subculture days. Scale bar = 0.5 cm. **b** Quantification parameters of AS regeneration ability of *MdAIL5*-OE lines and WT plants on different subculture days. Error bars = standard deviation. The same lowercase letter indicates no significant difference at *P* < .05 (Duncan’s multiple range test).

*AIL5* expression was significantly higher in young tissues than in mature tissues of *Arabidopsis* [47]. Correspondingly, *MdAIL5* expression in young apple tissues (flower bud, 30-day-old leaf, current-year shoot, and fruit core) was significantly higher than that in mature apple tissues (flower, 90-day-old leaf, perennial branch, and fruit flesh) (Fig. 2b). Further research found that *MdAIL5* expression in leaves decreased with an increase in subculture days of apple tissue culture seedlings (Fig. 2c). In addition to the genotype, the maturity of leaf explants is a crucial influencing factor for AS regeneration in apple [17]. In the current study, the AS regeneration ability of apple leaf explants decreased with an increase in the maturity of the explants. AS regeneration was difficult for ‘GL-3’ tissue cultured seedlings at 75 days after subculture (DAS). Conversely, the inhibitory effect of increased maturity of leaf explants was slightly less in the *MdAIL5*-OE lines. The AS increment coefficient, AS regenerative efficiency, and number of ASs per explant for the *MdAIL5*-OE lines were clearly higher than those for the WT plants (Fig. 3). The data indicated that *MdAIL5* overexpression can restore the AS regeneration ability of apple leaf explants to a certain extent, which was lost with an increase in maturity.

No remarkable morphological difference was observed between the *MdAIL5*-OE lines and non-transformed WT plants. However, some L11, L12, and L19 tissue-cultured seedlings produced numerous calli at the base of the seedlings and spontaneously formed adventitious roots (ARs) at nearly 60 DAS (Supplementary Data Fig. S2a). We further tested the effect of MdAIL5 on AR formation. The AR increment coefficient, AR regenerative efficiency, and number of ARs per explant for the *MdAIL5*-OE lines were higher than those for the WT plants (Supplementary Data Fig. S2b).

### *MdAIL5* overexpression changes apple hormone levels

**Figure 4:**
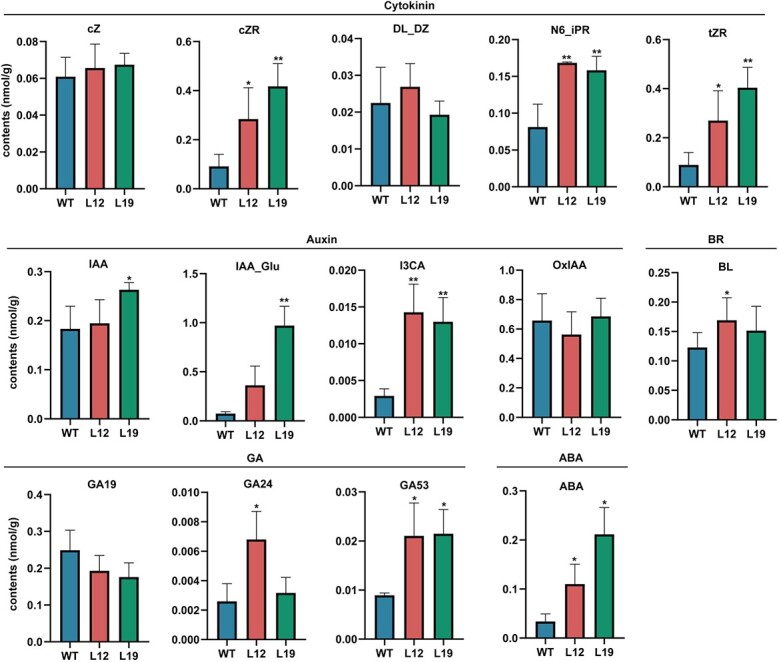
Contents of auxin, CK, GA, BR, and ABA in *MdAIL5*-OE lines and WT. Error bars = standard deviation. ^*^P < .05, ^**^P < .01 (Student’s *t*-test).

Auxin, CK, GA, BR, and ABA in *MdAIL5*-OE lines (L12 and L19) and WT were quantitatively detected through LC–MS/MS (Fig. 4). Among CK compounds, the cZ and DL_DZ content was not different between the *MdAIL5*-OE lines and WT. The cZR, N6_iPR, and tZR content was lower in WT than in the *MdAIL5*-OE lines, and the cZR and tZR content was higher in L19 than in L12. Among auxin compounds, the IAA_Glu and I3CA contents were lower in WT than in the *MdAIL5*-OE lines, and the IAA_Glu content was higher in L19 than in L12. The IAA content was higher in L19 than in L12 and WT. The OxIAA content was not clearly different between the *MdAIL5*-OE plants and WT. The BR content was slightly lower in WT than in L12 and L19. The ABA content was higher in the *MdAIL5*-OE lines than in WT. Among GA compounds, the GA19 content was higher in WT than in the *MdAIL5*-OE lines. By contrast, the GA53 content was lower in WT than in the *MdAIL5*-OE lines. In conclusion, *MdAIL5* overexpression causes significant changes in hormone content in apple.

**Figure 5:**
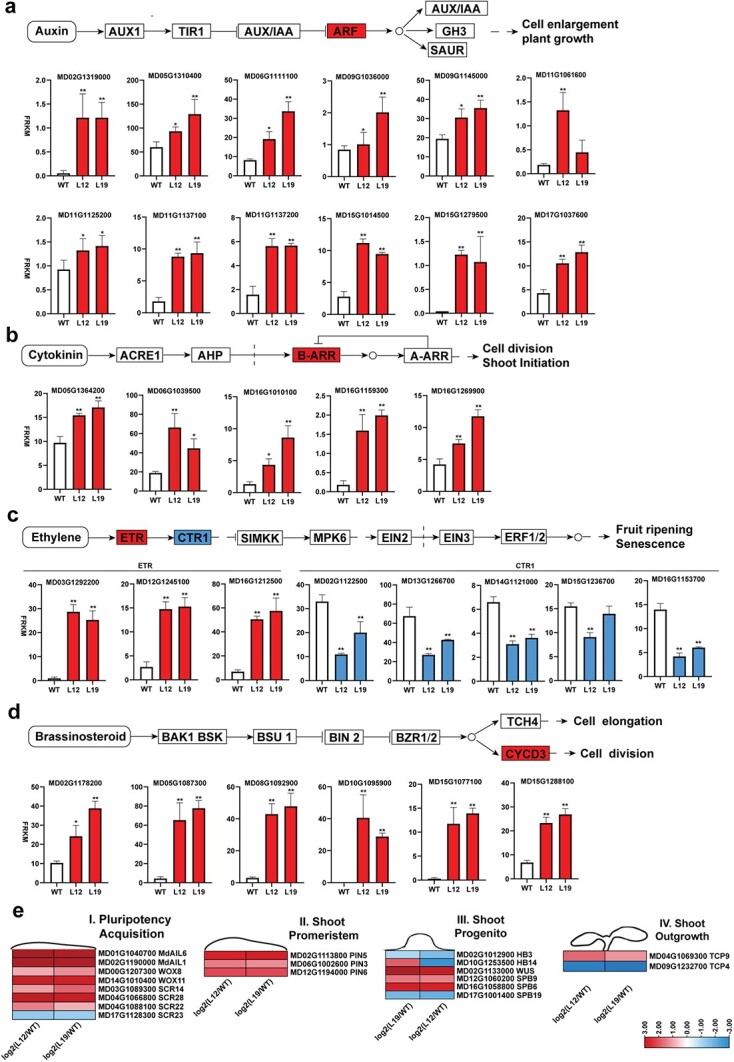
Expression of the phytohormone signal pathway and shoot development-related genes in *MdAIL5*-OE lines and WT. **a** Twelve *ARF* genes involved in auxin signaling were significantly downregulated in L12 and L19 versus WT. **b** Four *B-ARR* genes in the CK signaling pathway were significantly upregulated in L12 and L19 versus WT. **c** Three *ETR* genes and four *CTR1* genes in ETH signaling pathways were significantly upregulated and downregulated, respectively, in L12 and L19 versus WT. **d** Six *CYCD*s in BR signaling were upregulated in L12 and L19 versus WT. Error bars = standard deviation. ^*^Q < .05, ^**^Q < .01; *Q* value (adjusted *P* value) generated from DEG-seq [56]. Red and blue represent upregulation and downregulation, respectively. **e** Heat maps of DEGs (FPKM ≥2) related to different AS development stages. Red and blue represent upregulation and downregulation, respectively.

### *MdAIL5* overexpression changes expression levels of hormone- and shoot development-related genes

Genome-wide gene expression changes between the WT plants and *MdAIL5*-OE lines (L12 and L19) were analyzed through transcriptome analysis (RNA-seq) (Supplementary Data Fig. S3). In total, 1965 differentially expressed genes (DEGs) were detected when the L12 and WT libraries were compared, including 1154 upregulated DEGs and 811 downregulated DEGs. The 1965 DEGs were also detected when the L19 and WT libraries were compared, including 2218 upregulated DEGs and 1489 downregulated DEGs. In total, 602 DEGs upregulated and 149 DEGs downregulated in L12 were also upregulated and downregulated, respectively, in L19. Then, we performed GO classification and KEGG enrichment analysis to compare the functional differences in these DEGs with the same expression trend in L12 versus WT and L19 versus WT (Supplementary Data Fig. S4). The GO term ‘cellular anatomical entity’ belonged to the maximum number of genes among both upregulated and downregulated DEGs. Moreover, the KEGG enrichment analysis revealed that these DEGs were significantly enriched in hormone- and shoot development-related pathways.

**Figure 6:**
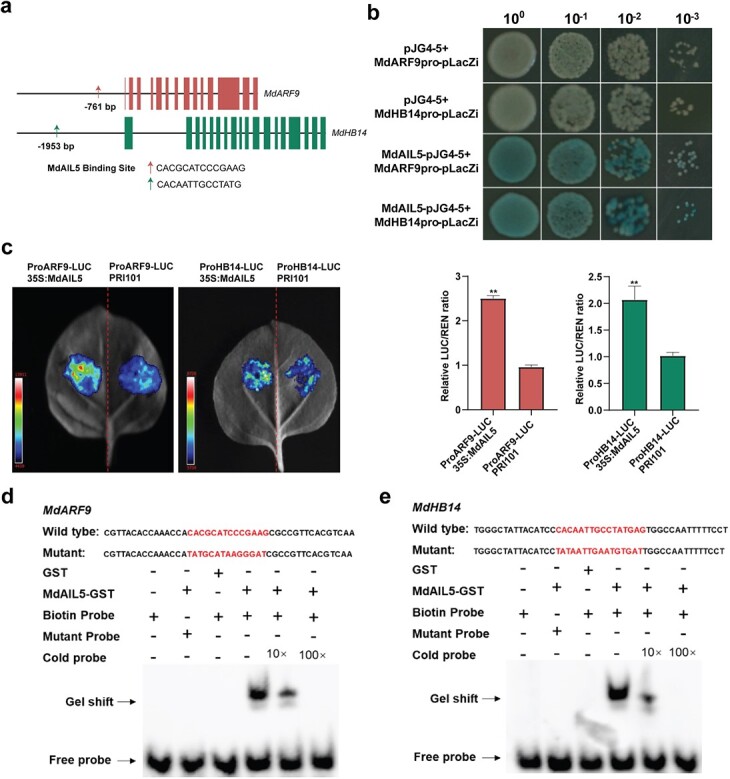
*MdARF9* and *MdHB14* are target genes of MdAIL5. **a** Schematic representation of the MdAIL5 binding site in *MdARF9* and *MdHB14* promoters. **b** Yeast-one-hybrid assay. MdAIL5-pJG4-5 constructs introduced with *MdARF9pro*-pLacZi and *MdHB14pro*-pLacZi separately into yeast strain EGY48. **c** Luciferase reporter assay. Luciferase intensity was measured. Error bars = standard deviation. ^**^P < .01 (Student’s *t*-test). **d** MdAIL5 binds to the CACGCATCCCGAAG motif in the *MdARF9* promoter. **e** MdAIL5 binds to the CACAATTGCCTATG motif in the *MdHB14* promoter.

*ARF* genes in auxin signaling are crucial regulators of plant AS regeneration [32]. In the present study, 12 upregulated *ARF* genes were detected in L12 versus WT and L19 versus WT. The expression levels of other auxin signaling-related genes, including *AUX1*, *TIR1*, *AUX*/*IAA*, *GH3*, and *SAUR*, were unchanged between the *MdAIL5*-OE lines and WT plants (Fig. 5a). In *Arabidopsis*, AIL/PLTs can induce YUCCA-mediated auxin accumulation. Song *et al*. [48] isolated 20 *YUCCA*s from the apple genome. Our transcriptome analysis revealed that three *MdYUCCA*s (*MdYUCCA10c*, *MdYUCCA4b*, and *MdYUCCA3a*) were significantly upregulated in both *MdAIL5*-OE lines (Supplementary Data Fig. S5). *YUCCA* encodes the rate-limiting enzyme for auxin biosynthesis. The increased auxin level in *MdAIL5*-OE line leaves may be due to the upregulation of these *YUCCA* genes. *A-ARR*s and *B-ARR*s positively and negatively regulate the CK response, respectively, and are involved in cell division and shoot initiation [27, 49]. Only upregulated *B-ARR*s were detected in transcriptome data, whereas no differentially expressed *A-ARR* was detected between the *MdAIL5*-OE lines and WT plants (Fig. 5b). *ETR* and *CTR1* are negative and positive regulators of AS regeneration, respectively [50]. The transcriptome analysis revealed that three ETR genes (MD13G1292200, MD12G1245100, and MD16G1212500) identified in the apple reference genome were clearly upregulated in the *MdAIL5*-OE lines. In contrast to ETR genes, five CTR1 genes (MD02G1122500, MD13G1266700, MD14G1121000, MD15G1236700, and MD16G1153700) were upregulated in the *MdAIL5*-OE lines (Fig. 5c). CYCDs are vital regulators of the plant BR response and positively regulate plant regeneration [51]. Six upregulated *CYCD* genes (MD02G1178200, MD05G1087300, MD08G1092900, MD10G1095900, MD15G1077100, and MD15G1288100) were detected in the two *MdAIL5*-OE lines (Fig. 5d).

Pluripotency acquisition, shoot promeristem formation, shoot progenitor formation, and shoot outgrowth are the four main stages of AS formation [34, 52–54]. *PLT*s, *WOX*s, and *SCR*s act as main pluripotency factors. In the current study, the expression levels of *MdAIL1* and 6, *WOX4*, *8*, and *11*, and *SCR14*, *22*, *28*, and *29* were clearly upregulated in both *MdAIL5*-OE lines. *PIN*s are indispensable for shoot promeristem formation. The expression levels of *PIN3*, *4*, *5*, and *6* were clearly upregulated in both *MdAIL5*-OE lines compared with the WT plants. AIL/PLTs regulate shoot-promoting *CUC* genes to establish the shoot promeristem in *Arabidopsis* [55]. We identified 13 *CUC*s from the apple reference genome (Supplementary Data Fig. S6b). The transcriptome analysis demonstrated that the expression of all *CUC* genes was unchanged in L12 and L19 compared with WT (Supplementary Data Fig. S6a and c). *HD-ZIP III*s, *SPL*s, and *WUS* are the main regulators in the shoot progenitor stage. *HB14*, *WUS*, and *SPB6* and *9* were significantly upregulated in the *MdAIL5*-OE lines. *TCP*s are indispensable for shoot outgrowth. *TCP9* was upregulated in both *MdAIL5*-OE lines, whereas *TCP4* and *TCP20* were downregulated (Fig. 5e). Moreover, based our published RNA-seq data, we analyzed the expression of hormone- and shoot development-related DEGs in apple leaves on 3, 7, 14, and 21 days after inoculation in the regeneration medium [21]. The results showed that these DEGs exhibited significant differential expression during AS regeneration (Supplementary Data Fig. S7).

### *MdARF9* and *MdHB14* are direct targets of MdAIL5 and are positively regulated by MdAIL5

To determine the target genes of MdAIL5, we analyzed whether the promoter regions of the aforementioned hormone- and shoot development-related DEGs contained MdAIL5-binding sites [57]. The promoter regions of *MdARF9* and *MdHB14* respectively contained two different MdAIL5-binding sites (Fig. 6a). The binding of MdAIL5 to the *MdARF9* and *MdHB14* promoters was determined through the Y1H assay (Fig. 6b). The electrophoretic mobility shift assay (EMSA) analysis confirmed this direct binding (Fig. 6d and e). The effector (MdAIL5) and reporter (*Luc* controlled by the *MdARF9* or *MdHB14* promoter) were co-injected into tobacco leaves, and the result showed that MdAIL5 clearly enhanced Luc/Ren activity (Fig. 6c).

### *MdARF9* and *MdHB14* overexpression enhances tobacco adventitious shoot regeneration

**Figure 7:**
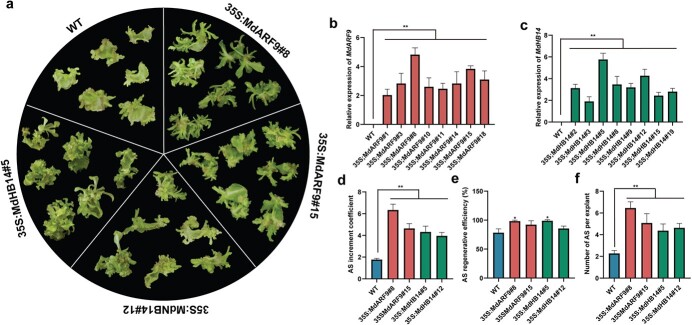
*MdARF9* and *MdHB14* overexpression enhanced tobacco AS formation. **a** Morphology of AS regenerated from the leaves of *MdARF9*-OE and *MdHB14*-OE tobaccos cultured on MS + 0.1 mg/l TDZ compared with non-transformed WT. **b** MdARF9 mRNA levels in *MdARF9*-OE tobaccos and WT. **c** MdHB14 mRNA levels in *MdHB14*-OE tobaccos and WT. **d**–**f** AS increment coefficient (**d**), AS regenerative efficiency (**e**), and number of ASs per explant (**f**) of *MdARF9*-OE tobaccos, *MdHB14*-OE tobaccos, and WT plants. Error bars = standard deviation. ^*^P < .05, ^**^P < .01 (Student’s *t*-test).

To analyze the function of MdARF9 and MdHB14 in AS regeneration, we generated *MdARF9*-OE and *MdHB14*-OE tobacco lines. No remarkable morphological changes were observed between the transgenic tobaccos and non-transformed WT plants. We detected MdARF9 and MdHB14 expression in eight randomly selected *MdARF9*-OE and *MdHB14*-OE tobacco lines, respectively (Fig. 7b and c). We then selected two transgenic tobacco lines with the highest expression levels of *MdARF9* (Fig. 7b) and MdHB14 (Fig. 7c) to compare their AS regeneration efficiencies with those of the WT tobacco lines. The results showed that the number of ASs per explant, AS increment coefficient, and AS regenerative efficiency were clearly higher in the *MdARF9*-OE and *MdHB14*-OE tobaccos than in the WT plants (Fig. 7a and d–f).

## Discussion

AS regeneration is a multistage developmental process. It includes the perception and transmission of plant hormone signals by somatic cells, initiation of cell division and proliferation, dedifferentiation with organ regeneration ability, and redifferentiation of organ formation [10, 34, 54]. An efficient AS regeneration system is the main factor leading to successful genetic transformation [7, 15]. Furthermore, the improvement in the AS regeneration efficiency of apple has a potential application in improving the asexual propagation of apple seedlings. Based on the crucial applied values, many studies have analyzed the AS regeneration mechanism of model plants such as *Arabidopsis*. However, the genetic basis for apple regeneration remains unclear.

Genotype is considered among the most critical factors affecting AS regeneration [7, 10, 16, 34]. However, unified identification of the AS regeneration efficiency of different apple genotypes is lacking. Therefore, we here conducted a unified identification of the AS regeneration ability of several apple genotypes. Among the identified cultivars and rootstocks, ‘Gala’ and M26 exhibited the highest AS regeneration efficiencies (Fig. 1). In addition, the expression of *MdAIL5*, which belongs to the AIL family, positively correlated with AS regeneration ability (Fig. 1b and c). Maturity of leaf explants is another main factor affecting AS regeneration efficiency [22]. The AS regeneration efficiency of apple decreased with increasing number of subculture days of tissue culture seedlings, and the *MdAIL5* mRNA levels were negatively correlated with leaf explant maturity (Fig. 2c and g). Studies have shown that *AIL* mRNA levels in the young leaves and stems of *Arabidopsis* are considerably higher than those in mature tissues [47, 58]. This expression characteristic of AIL5 is consistent between apple and *Arabidopsis* (Fig. 2b). The aforementioned findings indicated that the *MdAIL5* mRNA level is affected by genotype and explant maturity. MdAIL5 may be a key regulator of AS regeneration in apple.

Studies on *AIL5* have focused on its function in the regulation of germination and seed maturation [59–61], and little is known of its role in AS formation. To explore the role of *MdAIL5* in AS regeneration, we transformed the *MdAIL5* overexpression vector into the ‘GL-3’ apple. Then, the AS regeneration efficiencies of higher-expressing *MdAIL5*-OE lines (L11, L12, and L19) and WT plants on a regenerative medium with a low CK concentration were calculated. The results revealed that the AS formation efficiencies of the *MdAIL5-*OE lines were clearly higher than those of the ‘GL-3’ apple (Fig. 2f and g), which indicated that MdAIL5 positively regulates AS regeneration in apple. Notably, *MdAIL5* overexpression can restore the AS regeneration ability of apple leaf explants to a certain extent, which was lost with an increase in explant maturity (Fig. 3a and b). Studies have found that an increase in CK concentrations can restore the defect of the decline in the regeneration ability of adult plant leaves [22]. The recovery of AS regeneration ability of *MdAIL5*-OE highly mature explants may be related to the increase in the endogenous CK content.

The balance and crosstalk of auxin and CK determine the developmental fate of plant cells during *de novo* organogenesis [7, 8, 23, 34]. LC–MS/MS revealed that the CK and auxin levels increased in the *MdAIL5*-OE lines (Fig. 4). Transcriptome analysis revealed that *MdAIL5* overexpression changed the expression of hormone signaling pathway genes (Fig. 5a–d). *ARF*s, crucial mediators of auxin signaling, have key roles in AS formation. For example, *ARF5* can transactivate *STM* and *CRF2* promoters, thereby promoting callus formation and further improving the AS regeneration efficiency [32]. *ARF3* mutant explants could form a callus normally on CIM medium, but the number of ASs formed was significantly reduced when the calli were transferred to SIM [23]. Moreover, miR160 regulates *ARF10* expression, and the expression of main shoot regeneration genes, such as *WUS*, in transgenic explants expressing miR160-resistant ARF10 is significantly increased; the AS regeneration ability is significantly enhanced compared with the WT plants [34]. Upregulated expression of ARFs in the *MdAIL5*-OE lines suggests that there may be downstream genes of *MdAIL5* that promote AS regeneration in apple. B-ARRs, key regulators of the plant CK response, can directly bind to *WUS* and induce its expression, thereby significantly improving plant regeneration [27, 49]. In the current study, the mRNA levels of multiple *B-ARRs* in the *MdAIL5*-OE lines were clearly increased, indicating that *MdAIL5* can enhance the sensitivity of leaf explants to CK. Overall, the increased auxin and CK biosynthesis and sensitivity may play a major role in promoting AS regeneration of *MdAIL5*-OE lines.

Additional phytohormones, including ETH and BRs, are also vital for AS formation. Three upregulated *ETR* genes and five downregulated *CTR1* genes were detected in the *MdAIL5*-OE plants. Studies have found that ETH-related genes can positively and negatively regulate AS formation, determined by their role in ETH signaling. ETH-overproducing mutants and constitutive ETH response mutants exhibited an increased number of ASs, whereas ETH-insensitive mutants exhibited a decreased number of ASs in *Arabidopsis* [50]. The upregulated *ETR* genes and downregulated *CTR1* genes in the *MdAIL5*-OE lines were consistent with their positive and negative regulatory roles in AS formation, respectively. The *CYCD*s positively regulate cell division to promote plant regeneration [51]. The upregulated *CYCD*s detected in the present study indicated that they are key regulators of AS formation. Studies in model plants have shown that AS regeneration can be classified into pluripotency acquisition, shoot promeristem formation, establishment of the confined shoot progenitor, and shoot outgrowth, and specific family genes have a major regulatory role in each stage [34, 52–54]. Several genes involved in these stages, including *MdAIL6*, *MdPIN6*, *MdHB14*, and *TCP9*, were significantly and differentially expressed in the WT and *MdAIL5*-OE plants (Fig. 5e), which indicated that these genes act downstream of MdAIL5 to regulate AS regeneration at a specific stage. *Arabidopsis* AIL/PLTs promote AS regeneration through the activation of *CUC* genes [46]. We detected no *CUC* DEGs between the *MdAIL5*-OE lines and WT plants (Supplementary Data Fig. S6). Therefore, differences are observed in the mechanism of AIL5-mediated regulation of AS regeneration between apple and *Arabidopsis.*

AR formation is necessary for the vegetative propagation of horticultural crops such as apple [9, 62]. The differentiation and elongation of phloem parenchyma cells around the vascular bundles in the stem are the basis for the formation of AR primordia, and high IAA concentrations can induce this process [63, 64]. In *Arabidopsis*, the wounding-induced IAA peak regulates AR formation by activating *WOX11* expression [65]. In apple, MdWOX11 promotes AR formation by inducing *MdLBD29* expression [62]. In the current study, the increase in IAA content in the *MdAIL5*-OE lines was detected through LC–MS/MS (Fig. 4). In addition, transcriptome sequencing analysis found that *MdAIL5* overexpression induced *WOX11* expression (Fig. 4e, Supplementary Data Fig.S6). These two points may be the main reasons for the enhanced AR regeneration ability in *MdAIL5*-OE plants.

**Figure 8:**
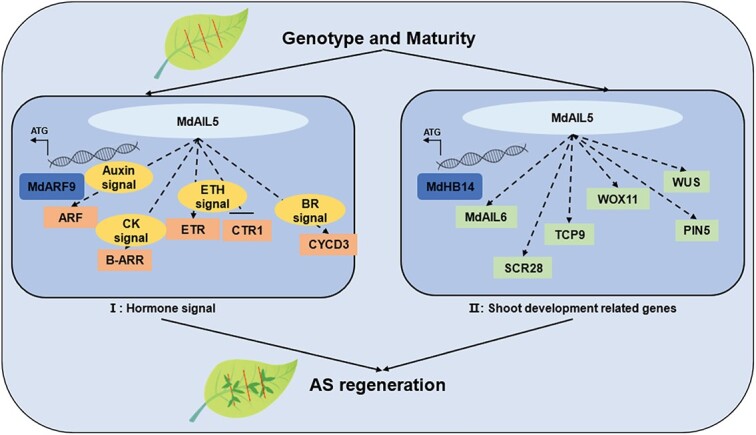
A hypothetical model of MdAIL5 regulating AS.

Y1H, EMSA, and dual-luciferase assays suggested that MdAIL5 could directly bind to the *MdARF9* and *MdHB14* promoters and trigger their expression to promote AS regeneration in apple (Fig. 6). Furthermore, *MdARF9* and *MdHB14* significantly improved the AS regeneration efficiency through stable genetic transformation of tobacco (Fig. 7). ARFs can activate or repress auxin response elements on their promoters [66]. Most studies on ARF9 have focused on the regulation of embryo and hypocotyl AR (HAR) formation. For example, Boutilier *et al*. [42] found that ARF9 can induce *LBD16* and *LBD28* expression by forming a protein complex with JMJ30, thereby promoting embryo development. ARF9 participates in the biogenesis of darkness-induced HARs by regulating *ARF7* and *ARF19* expression [67]. This is the first study to demonstrate that ARF9 plays a crucial role in promoting AS regeneration in plants. *MdHB14* belongs to HD-ZIP III. HD-ZIP III can bind to B-ARRs to form a transcriptional complex that upregulates *WUS*, thereby determining the spatial specificity of *WUS* expression [27]. Based on these results, we speculate that MdAIL5 promotes AS formation through two pathways (Fig. 8). (I) MdAIL5 activates the expression of *ARF*, *B-ARR*, *ETR*, and *CYCD3* and inhibits *CTR1* expression to regulate crossing among different hormone signaling pathways, thus ensuring signal integration for proper AS regeneration. (II) MdAIL5 can upregulate the expression of some shoot development-related genes, including *MdAIL6*, *MdSCR29*, *TCP9*, *WOX11*, *WUS*, and *PIN5*, to regulate AS formation at different stages. The genotype and maturity of leaf explants are critical factors affecting AS regeneration in apple. In addition, studies have found that B-ARRs can regulate *WUS* expression with the HD-ZIP III transcription factor forming a protein complex [27]. Thus, a relationship may exist between these two different regulation pathways, and this needs to be proven in our subsequent studies. Because AS regeneration is the crucial step of genetic transformation, clarifying the mechanism of MdAIL5-mediated promotion of AS regeneration lays a theoretical foundation for encouraging the application of genetic transformation in apple and other rosaceous fruit trees.

## Materials and methods

### Plant material

Tissue culture seedlings of ‘Gala’, ‘Golden Delicious’ (GD), ‘Hanfu’ (HF), ‘Fuji’, M16 × M9 (M26), Gm256, M9-T337 (T337), and B9 originated from buds collected from adult-phase materials in May. Tissue culture seedlings of ‘GL-3’ were provided by Prof. Zhihong Zhang (Shenyang Agricultural University, Shenyang, Liaoning). Tissue culture seedlings of the cold-hardy apple rootstocks BP-176, 54-118, and 71-3-150 were introduced from Michurinsk State Agricultural University, Michurinsk, Russia [68, 69]. All the tissue culture seedlings were cultivated at the Tissue Culture Center of the Institute of Pomology, Chinese Academy of Agricultural Sciences (40°37′N, 120°44′E). ‘Gala’, HF, GD, and ‘Fuji’ were cultured on MS medium with 0.3 mg/l 6-BA, 0.2 mg/l IAA, and 0.1 mg/l GA3. M26, T337, Gm256, B9, BP-176, 54-118, and 71-3-150 were cultured on MS medium with 0.8 mg/l 6-BA, 0.2 mg/l IAA, and 0.1 mg/l GA3. The tissue culture seedlings were cultured at 23°C under a 16-h photoperiod and transferred to a fresh medium every 6 weeks. The first four apical expanding leaves of the seedlings were transversely cut into 3- to 4-mm segments and used for AS regeneration. Four-week-old tissue culture seedlings were used for AR regeneration.

### Quantification parameters of adventitious shoot regeneration ability of apple leaves

AS regeneration ability was quantified by estimating the AS increment coefficient, regenerative efficiency, and number of ASs per explant. The results of three experiments were analyzed, with each experiment conducted using 60 explants. Quantification parameters of AS regeneration ability were calculated as follows:

AS increment coefficient = number of ASs/number of explants; AS regenerative efficiency (%) = (number of explants that regenerated ASs/number of explants) × 100%; and average number of ASs per explant = number of ASs/number of explants that regenerated ASs.

### Construction of vectors and transgenesis

The 1668-, 3107-, and 2000-bp coding sequences (CDSs) of *MdAIL5*, *MdARF9*, and *MdHB14* were amplified from the cDNA of ‘Gala’ leaves for the construction of the overexpression vector. Gene cloning was validated through sequencing. The CDSs were recombined into the plant expression vector PRI101 by using the ABclonal MultiF Seamless Assembly Mix (ABclonal Technology). Then, recombinant PRI101-MdAIL5, PRI101-MdARF9, and PRI101-MdHB14 were independently transformed into *Agrobacterium tumefaciens* EHA105. The primers are listed in Supplementary Data Table S1.

The genetic transformations of tobacco [70] and apple [12] are described elsewhere. ‘NC89’ and ‘GL-3’ were used as the genetic backgrounds for tobacco and apple transformations, respectively. The regenerated transgenic shoots of tobacco and apple were screened with 100 and 25 mg/l kanamycin monosulfate (Kan), respectively, after coculturing with *A. tumefaciens* EHA105 containing the specific recombinant plasmid. To maintain the selection pressure, the Kan-resistant shoots obtained from tobacco and apple were transferred to fresh medium every 3 weeks.

### Expression analysis by qRT–PCR

The primers for qRT–PCR were designed by Primer-BLAST software (Supplementary Data Table S1). cDNA was synthesized using the PrimeScript RT Regent Kit (TaKaRa). qRT–PCR was performed using TB Green Premix DimerEraser (TaKaRa) as described previously [21]. *MdActin* (MD12G1140800) was used as the endogenous reference gene, and gene relative expression level was calculated using the 2^−ΔΔ^ method [71].

### Hormone detection and analysis

The 100-mg samples for hormone extraction were harvested from the leaves of L11, L19, and WT. After the harvested sample was pretreated using a previously described procedure [72], the hormone was quantitatively detected through LC–MS/MS. In MultiQuant software (Sciex, USA), the default parameters were used for automatic identification and integration of each MRM transition (ion pair), and manual inspection was assisted [73].

### RNA-seq analysis

For transcriptome analysis, the first four apical expanding leaves of the tissue culture seedlings were collected for RNA extraction. Total RNA was extracted from three groups of leaves (WT, L12, and L19) by using the ethanol precipitation method and CTAB-PBIOZOL reagent, following the manufacturer’s instructions. Illumina RNA sequencing was then performed using the BGISEQ500 platform (BGI-Shenzhen, China), following previously described methods. The gene expression levels were calculated using the FPKM method [74]. Based on the criteria of fold change ≥2 and *Q* ≤ .05 (adjusted *P* ≤ .05), DEGs were identified using the DEGseq package [56]. The GO and KEGG enrichment analyses were performed using Phyper.

### Subcellular localization

The CDS of *MdAIL5* was recombined into PRI101-GFP by using the ABclonal MultiF Seamless Assembly Mix. The recombinant MdAIL5-pRI101-GFP was then transformed into *A. tumefaciens* GV3101 through heat shock. The resuspended solution was injected into the tobacco leaves by using a needle-free syringe and was cultured for 2 days before being placed on a glass slide. The resuspended solution was observed and photographed using a confocal scanning laser microscope (Leica TCS SP8, Leica, Germany). The primers are listed in Supplementary Data Table S1.

### Yeast one-hybrid analysis

The yeast one-hybrid (Y1H) analysis was performed as described elsewhere [9]. Through homologous recombination, the CDS of *MdAIL5* was recombined into pJG4-5 (AD-MdAIL5), and the promoter regions (2000 bp region upstream ATG) of *MdARF9* or *MdHB14* were recombined into pLacZi (BD-proMdARF9 or BD-proMdHB14, respectively). The plasmids were independently transformed into yeast (strain EGY48) [75]. We analyzed nine individual clones per experiment. The primers are listed in Supplementary Data Table S1.

### Transient transactivation assay

Transient transactivation was performed as described elsewhere [76]. Through homologous recombination, *MdARF9* and *MdHB14* promoters were recombined into pGreenII-0800-LUC (reporter constructs). The effector construct MdAIL5-PRI101 was prepared as described above. The treatment group was co-injected with a 1:1 (v:v) mixture of MdAIL5-PRI10 and proMdARF9-LUC or MdAIL5-PRI10 and proMdHB14-LUC, whereas the control group received a single injection of proMdARF9-LUC or proMdHB14-LUC. The primers are listed in Supplementary Data Table S1.

### Electrophoretic mobility shift assay

The CDS of *MdAIL5* was recombined into pGEX-4 T-1 containing the GST tag, and then the fusion construct was transformed into strain BL21. The MdAIL5–GST protein was expressed with 0.3 mM IPTG at 37°C for 8 h and purified using a Pierce™ GST Spin Purification Kit (Thermo Fisher Scientific). The *MdARF9* promoter fragment containing the sequence CACGCATCCAAG and the *MdHB14* promoter fragment containing the sequence CACAATTGCCTATG were labeled with biotin and used as probes, and the unlabeled ones were used as competitors. A LightShift™ Chemiluminescent EMSA Kit (Thermo Fisher Scientific) was used following the manufacturer’s instructions. The signals were captured using the ChemiDoc Imaging System (Bio-Rad). The primers are listed in Supplementary Data Table S1.

## Acknowledgements

This work was financially supported by the China Agriculture Research System (Grant No. CARS-27), the National Natural Science Foundation of China (Grant No. 32202463), and the Agricultural Science and Technology Innovation Program (Grant No. CAAS-ASTIP-2021-RIP -02). We would like to thank Prof. Zhihong Zhang (Shenyang Agricultural University, Shenyang, Liaoning) for providing tissue-cultured ‘GL-3’ plants. We would like to thank Prof. Jialong Yao (New Zealand Institute for Plant and Food Research Limited, Mount Albert Research Centre, Auckland, New Zealand) for providing the pGreenII-0800-LUC plasmid. We would like to thank Prof. Wei Li (China Agricultural University, Beijing) for providing pJG4-5 and pLacZi plasmids.

## Author contributions

C.X.Z., X.L.H., P.H.C., L.Y.Z. and K.L. conceived and planned the research. C.X.Z. and X.L.H. supervised the research. K.L., Z.L.L., A.Y., J.D.Y., P.H.C., X.L.H., G.P.Y. and C.X.Z. performed the experiments. K.L. and X.L.H. conducted data analysis. K.L. wrote the manuscript. C.X.Z. and X.L.H. edited the manuscript. All authors have read and agreed to the final version of the manuscript.

## Data availability

The data used to support the study findings are included within the article.

## Conflict of interest

The authors declare that they have no conflict of interest.

## Supplementary Material

Supplementary_Data_for_Review_uhad198

Figure_S1_uhad198

Figure_S2_uhad198

Figure_S3_uhad198

Figure_S4_uhad198

Figure_S5_uhad198

Figure_S6_uhad198

Figure_S7_uhad198
