# Supplementary material for: MdAIL5 overexpression promotes apple adventitious shoot regeneration by regulating hormone signaling and activating the expression of shoot development-related genes
Source: Hortic Res. 2023 Oct 10;10(11):uhad198. doi: 10.1093/hr/uhad198 (PMC10673654; doi:10.1093/hr/uhad198)
Supplement: Supplementary_Data_for_Review_uhad198 [file supplementary_data_for_review_uhad198.docx]

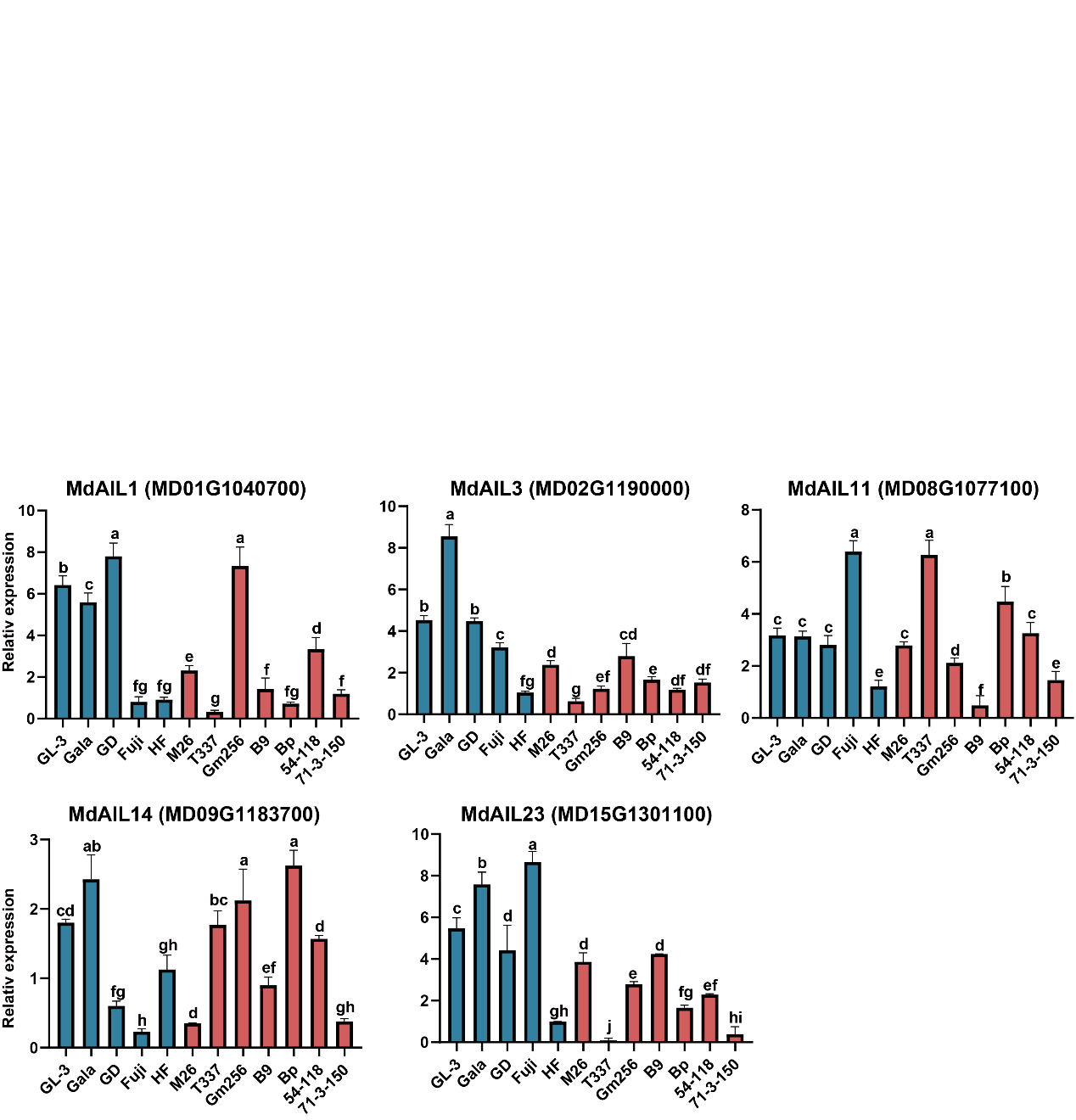


**Figure S1.** The relative expression of five *MdAILs* in apple cultivars and rootstocks. The data were collected from three independent experiments, with each experiment conducted using 60 explants. Error bars = SD. The same lowercase letter indicates no significant differences at level P < 0.05, Duncan’s multiple range test.


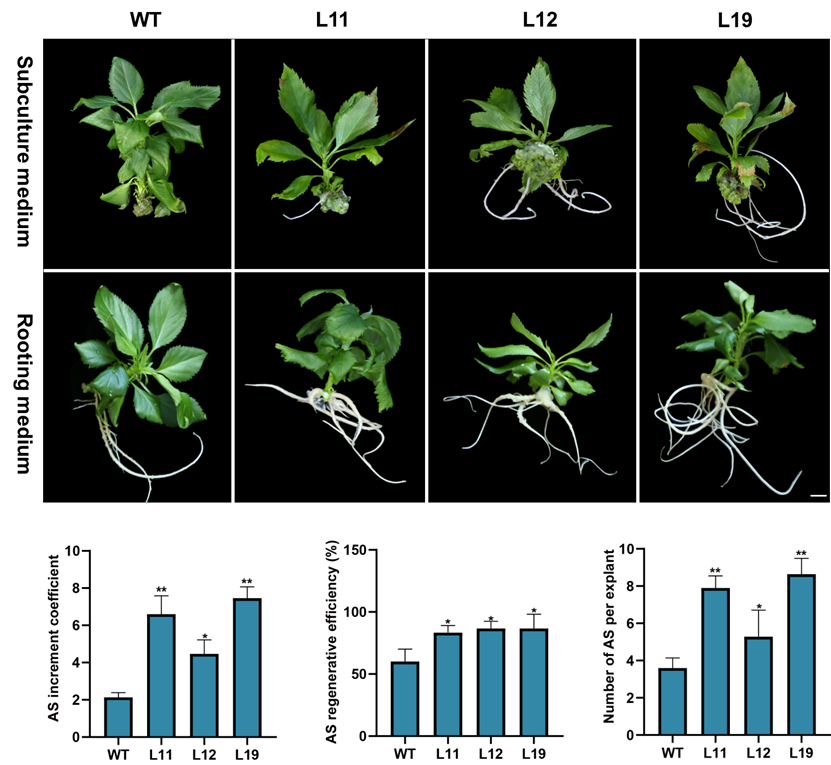


**Figure S2.** ***MdAIL5* overexpression enhanced apple AR regeneration.** a: Morphology of 60-day-old *MdAIL5*-overexpressing lines grown on subculture medium (MS with 0.3 mg/L. 6-BA, 0.2 mg/L IAA, and 0.1 mg /L GA3) and rooting medium (1/2 MS with 0.2 mg/L IBA), compared with the untransformed wild type (WT). Scalebar = 1 cm. b, c, and d represent the AR increment coefficient, AR regenerative efficiency, and number of AR per explant of MdAIL5-OE lines and WT plants, respectively. Error bars = SD. (Student’s t-tests, *P < 0.05, **P < 0.01).

**
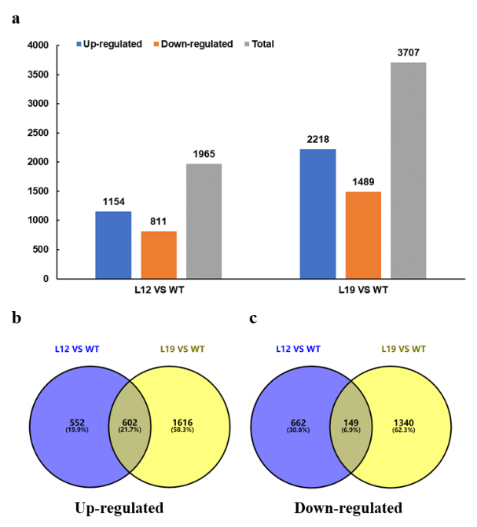
**

**Figure S3. DEGs identified by transcriptome analysis by comparisons of L12 and L19 to WT plants.** a: up- and down-regulated DEGs when MdAIL5-OE lines are compared with the WT; Venn plot of up-regulated (b) and down-regulated (c) DEGs in L12 and L19 compared with the WT.

**
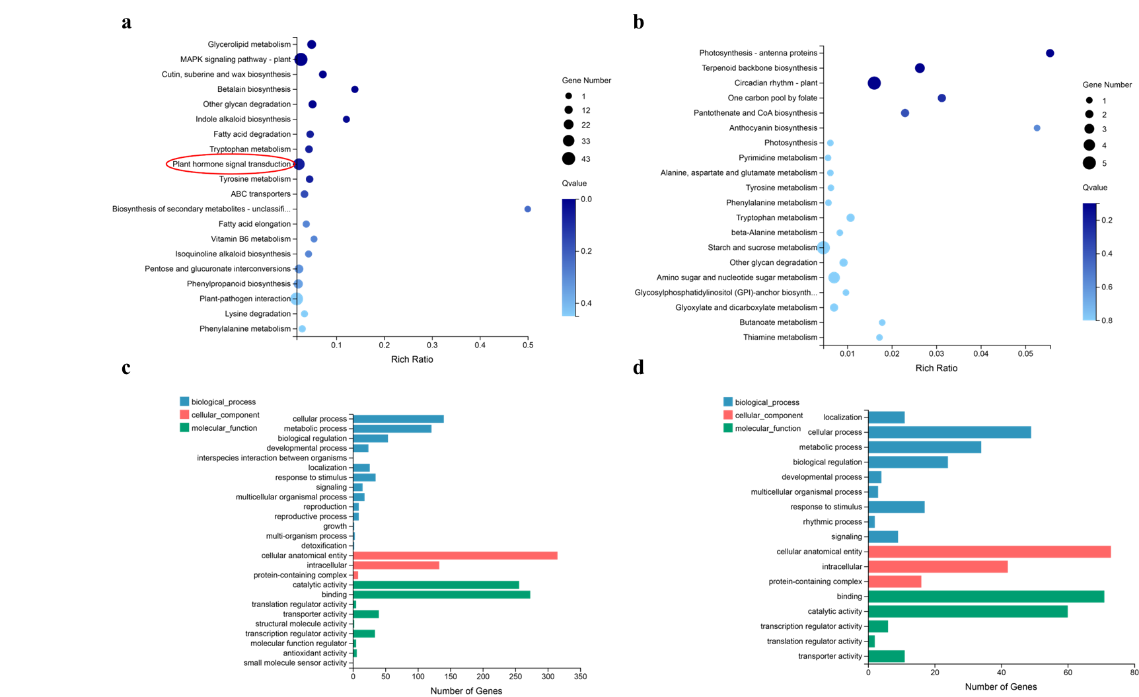
**

**Figure. S4 GO classification and KEGG enrichment analysis of DEGs.** a and b represent the KEGG pathway enrichment analysis of the upregulated and downregulated DEGs inMdAIL5-OE lines compared with WT, respectively. c and d represent the GO Classification analysis of the upregulated and downregulated DEGs in transgenic lines compared with WT plants, respectively. Only the top 15 terms with the smallest Q-value are shown.


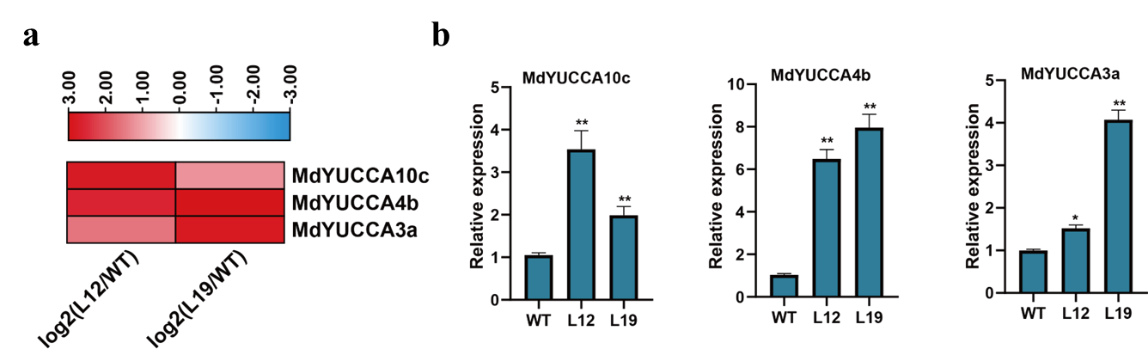


**Figure. S5.** Analyses of *YUCCA* genes. a: Heat map of *YUCCA* gene transcript levels in *MdAIL5*-OE lines compared with WT. Red and blue represent upregulation and downregulation, respectively. b: RT-qPCR detection of *YUCCA* gene transcript levels in *MdAIL5*-OE lines and WT. Error bars = SD. (Student’s t-tests, *P < 0.05, **P < 0.01).


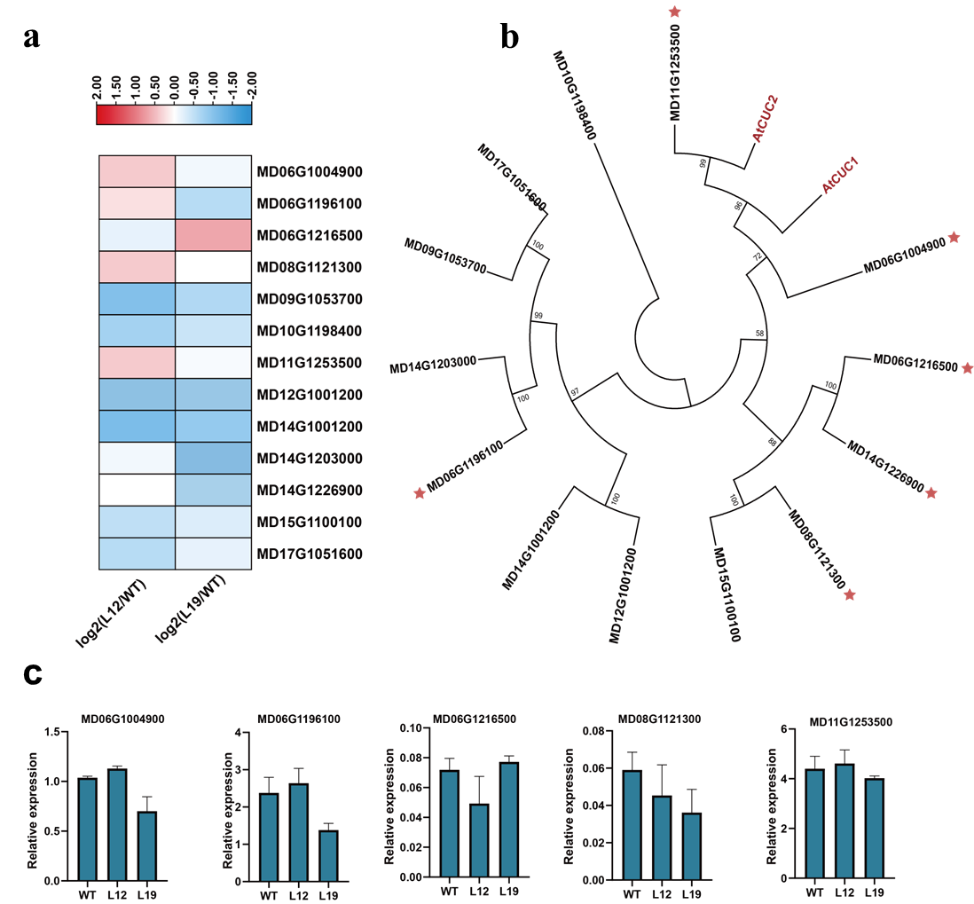


**Figure. S6.** Analyses of *CUP-SHAPED COTYLEDON* (*CUC*) genes. a: Heat map of *CUC* gene transcript levels in *MdAIL5*-OE lines compared with WT. Red and blue represent upregulation and downregulation, respectively. b: Phylogenetic tree of protein sequences of apple and Arabidopsis *CUC* proteins. The genes marked by the asterisk were up-regulated in *MdAIL5*-OE lines compared with the WT. c: qPCR detection of *CUC* gene transcript levels in *MdAIL5*-OE lines and WT.


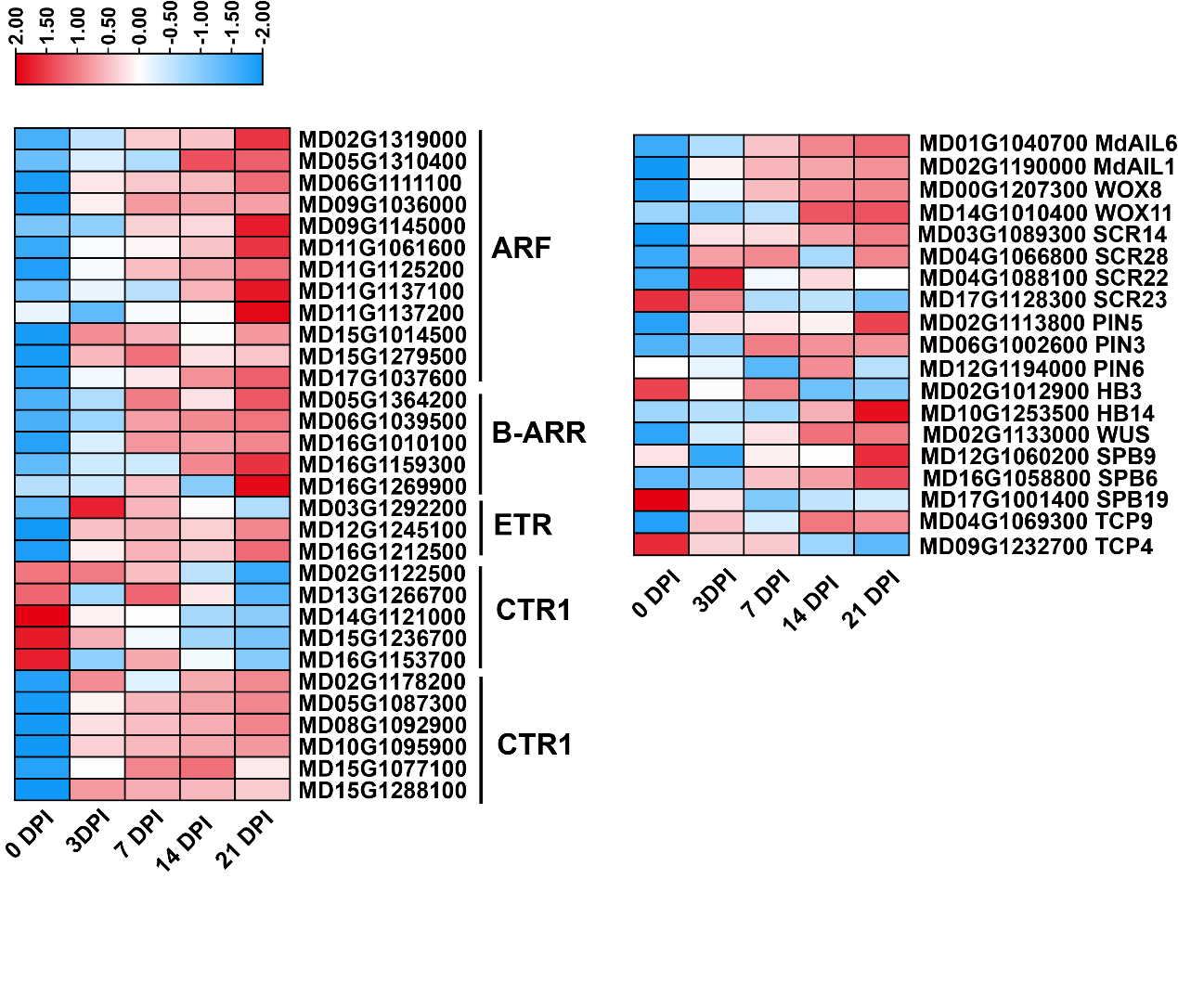


**Figure. S7.** Heat map of hormone and shoot development related DEGs transcript levels during AS regeneration (0, 3, 7, 14, and 21 DPI) of apple leaves. Red and blue represent upregulation and downregulation, respectively.

**Table S1.** Sequence of primers used for expression analysis, F for the former primer, R for the rear primer, MDP number of gene and length of primers.

| **Experiments** | **Primers** | **Sequence(5'→3')** |
| --- | --- | --- |
| **qRT-PCR** |  |  |
| *MdAIL1* | *MdAIL1*-qPCR-F | AGACTCGTCCCTGACTCACA |
|  | *MdAIL1*-qPCR-R | CTTTTTGGGCTTGAGGCACC |
| *MdAIL3* | *MdAIL3*-qPCR-F | CATCGGGTGTTATTGCTGCG |
|  | *MdAIL3*-qPCR-R | CCGTGGCAAAACTACGGTTG |
| *MdAIL5* | *MdAIL5*-qPCR-F | CCTTGGAACTTTCAGCACCCA |
|  | *MdAIL5*-qPCR-R | TAGCGGCTCATGTCGAAGTT |
| *MdAIL11* | *MdAIL11*-qPCR-F | TTCCGGTAGGGTTCTGCTCT |
|  | *MdAIL11*-qPCR-R | CGGCTGACTCCACGAAATCT |
| *MdAIL14* | *MdAIL14*-qPCR-F | TGGTTGTGGTGGTTGCTTCT |
|  | *MdAIL14*-qPCR-R | AGGGTTGGGGCTCACTTTTC |
| *MdAIL23* | *MdAIL23*-qPCR-F | TTCATGGGCAACGGCTACAT |
|  | *MdAIL23*-qPCR-R | CGCCGGACTATATGCAAGGT |
| *MdARF9* | *MdARF9*-qPCR-F | GGTAGTGGGTAGGATTAGGGG |
|  | *MdARF9*-qPCR-R | ACAGCATGGTGCAAACTGTC |
| *MdHB14* | *MdHB14*-qPCR-F | AGCAGGATATTTCATGGAGACTT |
|  | *MdHB14*-qPCR-R | TCGCATGCTTTTGGGAGAGA |
| *MdActin* | *MdActin*-qPCR-F | TGACCGAATGAGCAAGGAAATTACT |
|  | *MdActin*-qPCR-R | TACTCAGCTTTGGCAATCCACATC |
| MD01G1040700 | MD01G1040700-qPCR-F | GTCTTTCCAAGCTTGCAGCC |
|  | MD01G1040700-qPCR-R | TTGGTCAGGCTGTAACACCG |
| MD02G1133000 | MD02G1133000-qPCR-F | GATGGCTACTACTCCGGCTG |
|  | MD02G1133000-qPCR-R | GCAGCTAGCTTGCTAGAACTC |
| MD02G1319000 | MD02G1319000-qPCR-F | AAGAGCTTTTTGGACCTGGAGA |
|  | MD02G1319000-qPCR-R | ATGCCATCCTTGCGGTTTTG |
| MD03G1292200 | MD03G1292200-qPCR-F | CACGAGCAAGACCCTCCTAT |
|  | MD03G1292200-qPCR-R | GGAGGGCGAAAGACAGAGAC |
| MD05G1364200 | MD05G1364200-qPCR-F | GCCTCGTTTAGTGTCAGGGT |
|  | MD05G1364200-qPCR-R | AGCTAGCATTCATTTGTTGGACA |
| MD06G1111100 | MD06G1111100-qPCR-F | TGGTAGTGGGTAGGATTAGGGG |
|  | MD06G1111100-qPCR-R | CAGCATGGTGCAAACTGTCA |
| MD08G1092900 | MD08G1092900-qPCR-F | TCATCCCTCGCTAGTTCCGA |
|  | MD08G1092900-qPCR-R | CCACCTTCCCACAAGGATCG |
| MD10G1253500 | MD10G1253500-qPCR-F | TCATGGAGACTTTTCAGAGTGTT |
|  | MD10G1253500-qPCR-R | TGACACCGCGTGAAACCTAA |
| MD11G1061600 | MD11G1061600-qPCR-F | AGTTCTGGGATGGTGTCCCT |
|  | MD11G1061600-qPCR-R | AGCCCATCGTGTTTGTAGATG |
| MD14G1010400 | MD14G1010400-qPCR-F | CACTCCTCTGGACTCCCACT |
|  | MD14G1010400-qPCR-R | ACGAAAGACGATAACGAAACCAG |
| MD14G1121000 | MD14G1121000-qPCR-F | CGGACATCGTGAGGGTTAGG |
|  | MD14G1121000-qPCR-R | TTCTCTTCATCGGAGCCTCG |
| MD15G1014500 | MD15G1014500-qPCR-F | CCGAAACCCAGAGAATAGGATT |
|  | MD15G1014500-qPCR-R | TGAATTCGCTAAACGGGGCT |
| MD15G1288100 | MD15G1288100-qPCR-F | CTGTGGCATCACTGGACTCA |
|  | MD15G1288100-qPCR-R | CACGTCCACAGACACACGAT |
| MD16G1159300 | MD16G1159300-qPCR-F | CAATGCAAGGTGAGCCCATT |
|  | MD16G1159300-qPCR-R | TCACTTGCGCCGTGTAATCT |
| MD17G1002500 | MD17G1002500-qPCR-F | TGGTGTGGATCAAATGGTGCT |
|  | MD17G1002500-qPCR-R | TGCCCCTTAATTTGTGGAAAAACA |
| **Expression vector** |  |  |
| *MdAIL5-*pRI101(AN) | *MdAIL5*-*SalI*-PRI101-F | CACTGTTGATACATATGCCCGTCGACATGGATTCTTCTCCTCAGAACTGG |
|  | *MdAIL5*-*SalI*-PRI101-R | TCGGATCCGGTACCCCCGGGGTCGACTTATTCCATCCCAAAAATTGGTGT |
| *MdARF9-*pRI101(AN) | *MdARF9-SalI-PRI101-F* | CACTGTTGATACATATGCCCGTCGACATGGCGAATCGGGAAGG |
|  | *MdARF9*-*SalI*-PRI101-R | TCGGATCCGGTACCCCCGGGGTCGACTTAGTCCGAGCTTATTACCGGTC |
| *MdHB14-*pRI101(AN) | *MdHB14*-*SalI*-PRI101-R | TCGGATCCGGTACCCCCGGGGTCGACTTAGTCCGAGCTTATTACCGGTC |
|  | *MdHB14-SalI*-PRI101-F | CACTGTTGATACATATGCCCGTCGACATGGCGCTGGTAATGCAC |
| **Y1H** |  |  |
| MdAIL5-CDS-pJG4-5 | MdAIL5-EcoRI-pJG4-5-F | TGCCAGATTATGCCTCTCCCGAATTCATGGATTCTTCTCCTCAGAACTGG |
|  | MdAIL5-EcoRI-pJG4-5-R | CAAAGCTTCTCGAGTCGGCCGAATTCTTATTCCATCCCAAAAATTGGTGT |
| *MdARF914*-Promoter-placZi | *MdARF9pro*- SalI-placZi-F | TCGGATCCGGTACCCCCGGGGTCGACTTAGTCCGAGCTTATTACCGGTC |
|  | *MdARF9pro*- SalI-placZi-R | CACTGTTGATACATATGCCCGTCGACATGGCGCTGGTAATGCAC |
| *MdHB14*-Promoter-placZi | *MdHB14pro*- SalI-placZi-F | AGCTCGGTACCCGGGGATCTGTCGACCACCCATTGGAAGATGGGC |
|  | *MdHB14pro*- SalI-placZi-R | TACAGAGCACATGCCTCGAGGTCGACTGTACGGACGCAACAAAACAAA |
| **Transient transactivation** |  |  |
| *MdAIL5-*pRI101-GFP(AN) | *MdAIL5*-*SalI*-PRI101-GFP-F | CACTGTTGATACATATGCCCGTCGACATGGATTCTTCTCCTCAGAACTGG |
|  | *MdAIL5*-*SalI*-PRI101-GFP-R | TCGGATCCGGTACCCCCGGGGTCGACTTCCATCCCAAAAATTGGTGT |
| *MdARF9*-Promoter- pGreenII 0800-LUC | *MdARF9pro*-*BamHI*-0800-F | TCGAATTCCTGCAGCCCGGGGGATCCTCTGGGGAAAAGAAAAGGTGAT |
|  | *MdARF9pro*-*BamHI*-0800-R | GCGGCCGCTCTAGAACTAGTGGATCCCGTTGAACTCCAACCCTCC |
| *MdHB14*-Promoter- pGreenII 0800-LUC | *MdHB14pro*-*BamHI*-0800-F | TCGAATTCCTGCAGCCCGGGGGATCCCACCCATTGGAAGATGGGC |
|  | *MdHB14pro*-*BamHI*-0800-R | GCGGCCGCTCTAGAACTAGTGGATCCTGTACGGACGCAACAAAACAAA |
| **Probe for EMSA** |  |  |
| *MdARF9*-Promoter | *MdARF9pro-*F Hot probe | TGGGCTATTACATCCCACAATTGCCTATGAGTGGCCAATTTTTCCT |
|  | *MdARF9pro-*R Hot probe | AGGAAAAATTGGCCACTCATAGGCAATTGTGGGATGTAATAGCCCA |
|  | *MdARF9pro-*F Cold probe | TGGGCTATTACATCCCACAATTGCCTATGAGTGGCCAATTTTTCCT |
|  | *MdARF9pro-*R Cold probe | AGGAAAAATTGGCCACTCATAGGCAATTGTGGGATGTAATAGCCCA |
|  | *MdARF9pro-*F Mutant cold probe | TGGGCTATTACATCCTATAATTGAATGTGATTGGCCAATTTTTCCT |
|  | *MdARF9pro-*R Mutant cold probe | AGGAAAAATTGGCCAATCACATTCAATTATAGGATGTAATAGCCCA |
| MdHB14-Promoter | *MdHB14pro-*F Hot probe | CGTTACACCAAACCACACGCATCCCGAAGCGCCGTTCACGTCAA |
|  | *MdHB14pro-*R Hot probe | TTGACGTGAACGGCGCTTCGGGATGCGTGTGGTTTGGTGTAACG |
|  | *MdHB14pro-*F Cold probe | CGTTACACCAAACCACACGCATCCCGAAGCGCCGTTCACGTCAA |
|  | *MdHB14pro-*R Cold probe | TTGACGTGAACGGCGCTTCGGGATGCGTGTGGTTTGGTGTAACG |
|  | *MdHB14pro-*F Mutant cold probe | CGTTACACCAAACCATATGCATAAGGGATCGCCGTTCACGTCAA |
|  | *MdHB14pro-*R Mutant cold probe | TTGACGTGAACGGCGATCCCTTATGCATATGGTTTGGTGTAACG |
