# Supplementary figures and images for: MdAIL5 overexpression promotes apple adventitious shoot regeneration by regulating hormone signaling and activating the expression of shoot development-related genes
Source: Hortic Res. 2023 Oct 10;10(11):uhad198. doi: 10.1093/hr/uhad198 (PMC10673654; doi:10.1093/hr/uhad198)

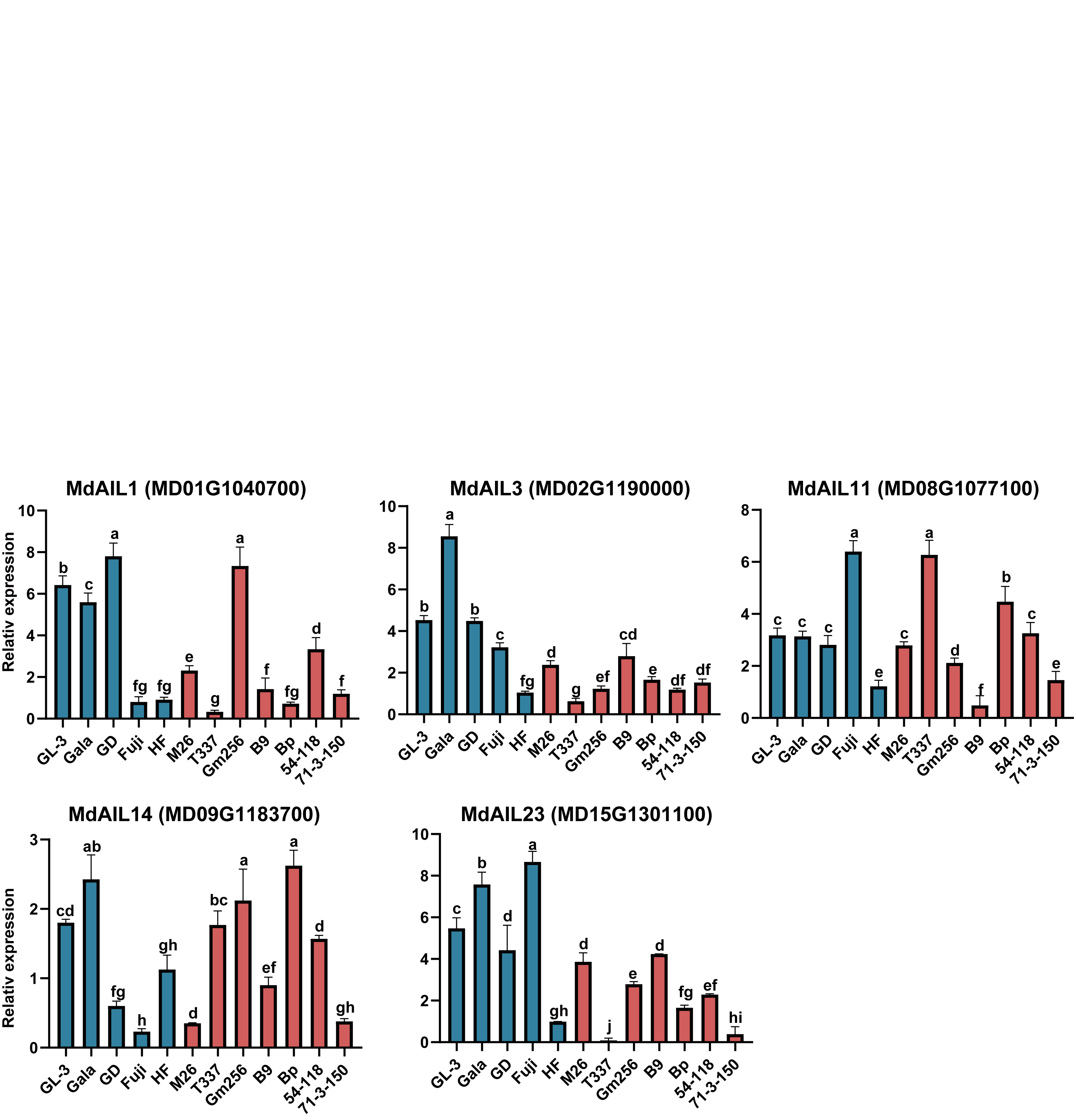

Supplement: Figure_S1_uhad198 [file figure_s1_uhad198.zip › Figure_S1_uhad198.tif]

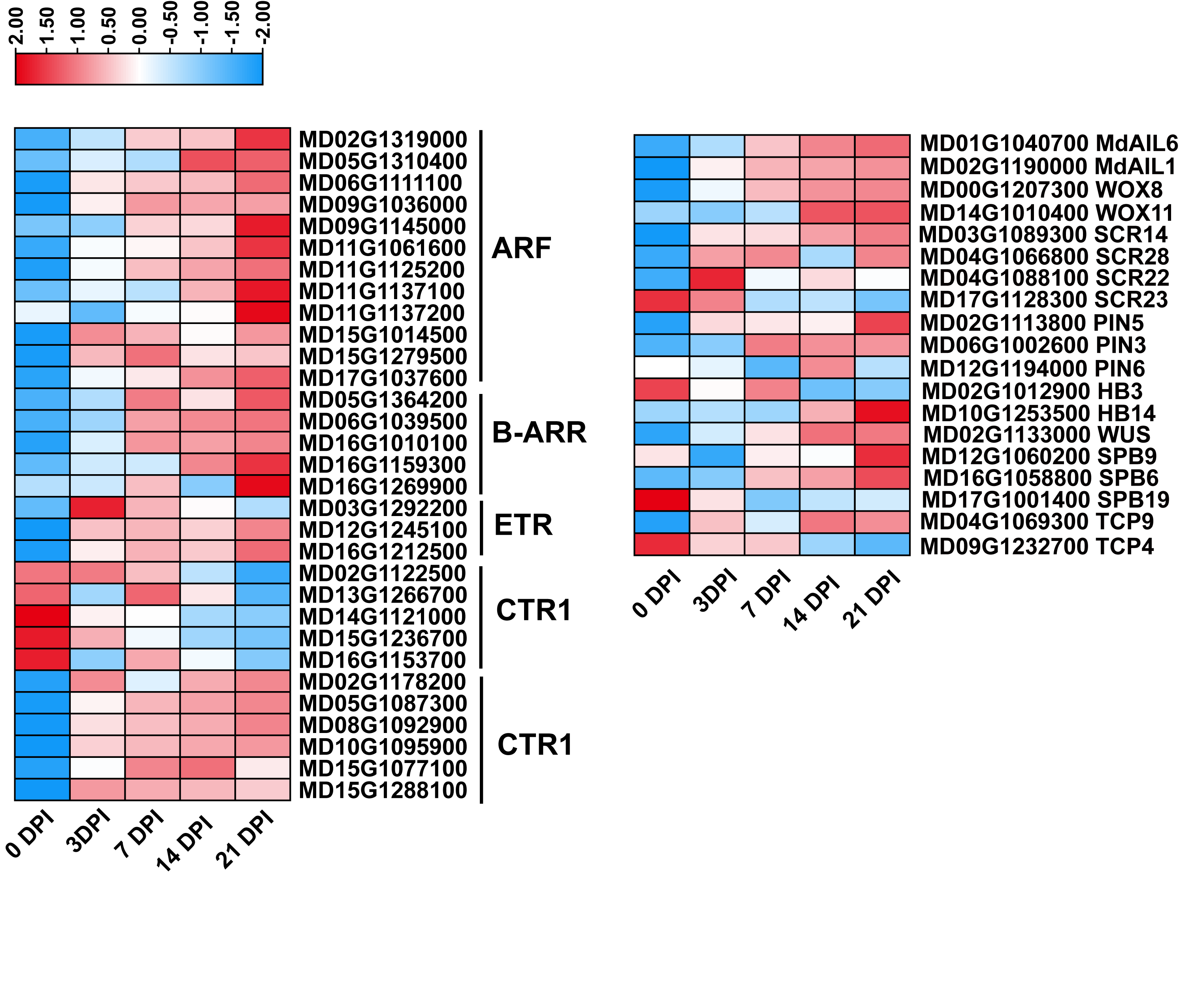

Supplement: Figure_S7_uhad198 [file figure_s7_uhad198.zip › Figure_S7_uhad198.tif]
